# Editorial: Molecular imaging in retinal diseases

**DOI:** 10.3389/fmed.2023.1180330

**Published:** 2023-03-28

**Authors:** Seong Joon Ahn

**Affiliations:** Department of Ophthalmology, Hanyang University Hospital, Hanyang University College of Medicine, Seoul, Republic of Korea

**Keywords:** biomarker, molecular imaging, retinal disease, age-related macular degeneration, retinal imaging

Retinal diseases, including a wide variety of diseases, such as age-related macular degeneration, diabetic retinopathy, retinal detachment, epiretinal membrane, and retinitis pigmentosa, can be caused by diverse pathogenic processes, such as aging, angiogenesis, infections/inflammation, trauma, drug-induced toxicity, and genetic mutation. Visual impairment secondary to retinal diseases is a global public health problem that can be a significant economic burden to society due to the loss of labor productivity. Therefore, early diagnosis and appropriate management of these diseases are required.

Advances in clinical imaging have enabled visualization of the retina at unprecedented resolution. For example, optical coherence tomography (OCT) provides non-invasive, quasi-histological cross-sectional images of the retina, which have revolutionized the diagnosis of retinal diseases. However, most imaging modalities detect the disease after the structural changes caused by diverse pathological processes become apparent. Early detection of retinal diseases is an unmet clinical need, which may allow for early intervention and better outcomes. In this light, molecular imaging has provided a window into dynamic pathogenic processes using molecular biomarkers in human diseases (1). By visualizing pathological processes at molecular and cellular levels, imaging might enable earlier detection of diseases before overt structural changes (1, 2). Furthermore, molecular imaging has a great potential to improve patient outcomes by applying targeted therapy and measuring molecular-specific effects of treatment.

Recently, advances have been made in the field of molecular imaging of retinal diseases. These have shown promising results for the early detection of retinal diseases. For example, several retinal endothelial cell biomarkers may visualize choroidal neovascularization, a hallmark of age-related macular degeneration, at a stage before which it can be detected using conventional imaging, such as fluorescein angiography (3–5). Accordingly, many endothelial cell-targeted therapies are in development to make possible combined imaging and therapy of retinal disease, which is also known as theranostics.

Furthermore, artificial intelligence (AI) is increasingly being applied to retinal diseases, and retinal image analysis using AI has demonstrated significant potential for the screening of retinal diseases, such as diabetic retinopathy, age-related macular degeneration, and retinopathy of prematurity (6–8). This may also reveal novel, important biomarkers that have not yet been identified by clinicians or researchers, which may have clinical applications for diagnostic and prognostic purposes.

In this Research Topic, three original research articles report the development and verification of molecular imaging techniques or animal models of retinal diseases. In a mouse model of age-related macular degeneration, Papuaga et al. generated a novel optical imaging probe to visualize NLRP3 inflammasomes in choroidal neovascularization lesions and proposed that the probe may be a useful molecular imaging tool to monitor the onset, progression, and therapeutic response of age-related macular degeneration. Jeon et al. demonstrated the imaging of retinal CX3CR1-GFP+ cells, which were useful in analyzing the behavior of inflammatory cells after arterial recanalization in an animal model of branch retinal artery occlusion. Yang et al. developed a novel knockout model of retinitis pigmentosa using *Pde6b*-knockout Long–Evans rats, which showed characteristic retinal features. Serial fundus images and OCT of *Pde6b*-knockout Long–Evans rats showed bone spicule pigmentation and progressive retinal thinning, respectively.

Kuang et al. developed segmentation methods for retinal vessels, which can be important biomarkers for systemic diseases (e.g., cardiovascular diseases). The methods combined unsupervised methods of local phase congruency and orientation scores with the deep-learning network modified from U-Net, which resulted in superior performance compared with conventional methods, particularly for small vessel segmentation. Finally, a review article by Yusuf et al. summarized novel retinal imaging techniques potentially applicable to screening hydroxychloroquine-induced retinal toxicity. The techniques included en-face imaging, topographic thickness maps, sequential retinal thickness analysis, fluorescence lifetime imaging ophthalmoscopy, near-infrared fundus autofluorescence, and quantitative autofluorescence. Better or earlier detection of the disease at the preclinical stage using novel imaging techniques and AI-assisted diagnostics may further reduce the risk of visual loss due to retinal toxicity or other causes.

In summary, this Research Topic focused on the identification of novel biomarkers and advances in molecular imaging techniques for retinal diseases. These articles highlight progress in the current understanding of the pathogenesis of several common retinal diseases and the potential of molecular imaging techniques for earlier diagnosis of the diseases. These studies provide insights into future translational research and clinical applications of molecular imaging with the final aim of improving the outcome of the disease by resolving the unmet needs of early detection.

## Author contributions

The author confirms being the sole contributor to this study and has approved it for publication.
